# RETRACTED: Gupta et al. Self-Gelling Solid Lipid Nanoparticle Hydrogel Containing Simvastatin as Suitable Wound Dressing: An Investigative Study. *Gels* 2022, *8*, 58

**DOI:** 10.3390/gels11080640

**Published:** 2025-08-13

**Authors:** Bhumika Gupta, Garima Sharma, Pratibha Sharma, Simarjot Kaur Sandhu, Indu Pal Kaur

**Affiliations:** University Institute of Pharmaceutical Sciences, Panjab University, Chandigarh 160014, India; bhumika.gupta30@gmail.com (B.G.); garimasharma194@gmail.com (G.S.); pratibhapulastya@gmail.com (P.S.); simarjots@gmail.com (S.K.S.)

The journal retracts the article titled “Self-Gelling Solid Lipid Nanoparticle Hydrogel Containing Simvastatin as Suitable Wound Dressing: An Investigative Study” [1], cited above.

Following publication, concerns were brought to the attention of the publisher regarding image overlap contained within Figure 11 in [1].

Adhering to our standard procedure, an investigation was conducted by the Editorial Office and the Editorial Board that confirmed the presence of image duplications within the control group of Figure 11, presenting different experimental conditions. While the authors collaborated with the Editorial Office during the investigation, they were unable to provide supporting raw material for Editorial Board evaluation that meets the journal’s original image requirements (https://www.mdpi.com/journal/gels/instructions#oriimages). As a result, the Editorial Board lost confidence in the reliability of the findings and decided to retract this article [1] as per MDPI’s retraction policy (https://www.mdpi.com/ethics#_bookmark30).

This retraction was approved by the Editor-in-Chief of the *Gels* journal.

The authors disagree to this retraction.
